# PHACK: An Efficient Scheme for Selective Forwarding Attack Detection in WSNs

**DOI:** 10.3390/s151229835

**Published:** 2015-12-09

**Authors:** Anfeng Liu, Mianxiong Dong, Kaoru Ota, Jun Long

**Affiliations:** 1 School of Information Science and Engineering, Central South University, ChangSha 410083, China; afengliu@mail.csu.edu.cn; 2 Department of Information and Electronic Engineering, Muroran Insitute of Technology, Hokkaido 050-8585, Japan; mx.dong@csse.muroran-it.ac.jp (M.D.); ota@csse.muroran-it.ac.jp (K.O.)

**Keywords:** wireless sensor networks, selective forwarding attack, malicious node, network lifetime

## Abstract

In this paper, a Per-Hop Acknowledgement (PHACK)-based scheme is proposed for each packet transmission to detect selective forwarding attacks. In our scheme, the sink and each node along the forwarding path generate an acknowledgement (ACK) message for each received packet to confirm the normal packet transmission. The scheme, in which each ACK is returned to the source node along a different routing path, can significantly increase the resilience against attacks because it prevents an attacker from compromising nodes in the return routing path, which can otherwise interrupt the return of nodes’ ACK packets. For this case, the PHACK scheme also has better potential to detect abnormal packet loss and identify suspect nodes as well as better resilience against attacks. Another pivotal issue is the network lifetime of the PHACK scheme, as it generates more acknowledgements than previous ACK-based schemes. We demonstrate that the network lifetime of the PHACK scheme is not lower than that of other ACK-based schemes because the scheme just increases the energy consumption in non-hotspot areas and does not increase the energy consumption in hotspot areas. Moreover, the PHACK scheme greatly simplifies the protocol and is easy to implement. Both theoretical and simulation results are given to demonstrate the effectiveness of the proposed scheme in terms of high detection probability and the ability to identify suspect nodes.

## 1. Introduction

Wireless Sensor Networks (WSNs) are emerging as one of the prevailing technologies of the future due to their wide range of applications in military and civilian domains [1,2,3,4,5,6,7,8,9]. Due to their operating nature, they are often unattended and hence prone to different types of novel attacks [3,4,5,10]. For instance, an adversary could capture nodes, acquiring all the information stored therein—sensors are commonly assumed to not be tamper-proof [3,4,5,10]. Therefore, an adversary may replicate captured sensors and deploy them in the network to launch a variety of malicious activities [10]. Because the decisions made by a sensor network depend on the data collected by the sensor nodes, if an attacker prevents the important or sensitive data from being forwarded to the sink, this will cause the complete failure of the network or result in the wrong decision being made, potentially causing serious damage [10,11]. The selective forwarding attack (SFA) is one such attack [3,4,5]. According to the research, many attack behaviors in sensor networks are related to the selective forwarding attack, such as the DoS attack, which is equivalent to a very serious selective forwarding attack behavior in which all received packets are discarded. What makes things worse is that the SFA is very simple to implement but is a difficult attack behavior to detect. In the SFA, a malicious node works as a normal node but refuses to forward certain selected packets and instead simply drops them. Thus, due to this nature, the SFA is very harmful for mission critical applications and can damage the overall network communication, making the network useless [5]. Therefore, how to detect and avoid the selective forwarding attack is meaningful for wireless sensor network security [3,4,5].

In this paper the Per-Hop Acknowledgement (PHACK)-based SFA detection scheme, which can effectively detect selective forwarding attacks and recover dropped data is proposed. The innovative points of this paper are mainly:

(1) The PHACK scheme proposed in this paper has better ability for detecting and identifying suspect nodes. In the PHACK scheme, each intermediate node along a forwarding path is responsible for generating acknowledgements (ACK) to the source node for each packet received. The difference from previous research is that each confirmation is routed to the sink along a different path. One benefit is that the probability of the confirmation information reaching the sink successfully can be improved, as this approach can avoid the risk of a single routing failure for the case in which all ACK packets are returned to the source node along the same data forwarding path as in previous work; additionally, because each acknowledgement is independently routed along different paths, this scheme has a higher ability for detecting and identifying suspect nodes [3,4,5]. Though the acknowledgement can be returned by each intermediate node in this scheme, the network lifetime was not affected in comparison to other schemes. This is because, in the process of data collection in a wireless sensor network, the nodes near the sink consume more energy due to the increased amount of data that can be forwarded to the sink from the nodes far from the sink, called hotspots. After the premature death of nodes near the sink area forms an energy hole, the data from nodes in the distance cannot be routed to the sink, which causes the entire network to die in advance [8], with more than 90% of the total energy being unable to be used [12]. This means that if the residual energy can be used effectively, it not only does not affect the network lifetime but also can improve the ability for detecting the selective forwarding attack. Theoretical analysis has proved that although the PHACK scheme increases the energy consumption in the peripheral area, it does not increase the energy consumption in hotspot areas, so the network lifetime in the PHACK scheme is not less than that of other acknowledgement-based schemes, but the performance can be improved significantly with regard to the detection accuracy and effectiveness.
(2) Second, the PHACK scheme can not only effectively detect the selective forwarding attack, but it also has the ability to recover from routing failure, as the attacked data can be rerouted to the sink rapidly along an alternative routing path that excludes the suspect nodes. In the previous research, most of the selective forwarding attack detection schemes only have a detection function. In the PHACK scheme, suspect nodes can be accurately identified, and thus the dropped data can be rerouted from the nodes nearest to the sink to the sink along a routing path that bypasses the suspect nodes; this leads to the quick recovery of the routing data at the lowest cost.
(3) The performance of the PHACK scheme has been proved through theory analysis and extensive simulation results via Omnet++. The validity of this scheme can be confirmed by the experimental results. In the contrast experiment, the detection probability and the veracity of the identification of suspect nodes are better than in previous studies.

The rest of this paper is organized as follows: in Section 2, related works are reviewed. The system model is described in Section 3. In Section 4, a novel selective forwarding attack detection integrated routing recovery scheme is presented. Security analysis and performance analysis are provided in Section 5. The analysis and comparison of the experimental results are shown in Section 6. We conclude in Section 7.

## 2. Related Work

Because selective forwarding attacks can cause great harm to a network, there have been various research studies regarding the detection and avoidance of such attacks. The related important works are summarized in this section. Due to the good performance of the acknowledgement-based selective forwarding attack detection scheme, we mainly discuss this scheme in this section. Implementations of the acknowledgement-based SFA detection scheme can be divided into the following categories:

(1) Early schemes did not detect whether there is an SFA, but rather adopted multiple routes to ensure that the data reaches the sink at a higher probability when the network is attacked [3,13]. This is effective for most attacks that prevent data from being transmitted to the sink [3,13]. However, this scheme does not detect whether an SFA exists and also does not have the ability to identify malicious nodes [3,13]. At the same time, the communication overhead and energy consumption are huge, as they are proportional to the number of paths. Karlof *et al.* [13] firstly discuss the selective forwarding attack and also suggest that multi-path routing can be used to counter these types of attacks.
(2) Subsequent schemes can detect whether there an SFA exists, but cannot identify malicious nodes. The main point of this type of scheme is to detect whether an SFA exists in the process of data transmission; once an SFA is identified in the process, the scheme sends a warning message to the system. However, this type of scheme does not have the ability to identify malicious nodes [3]. Sun *et al.* [14] proposed a multi-dataflow topology (MDT) method to countermeasure the selective forwarding attacks. In an MDT, if there is a malicious node in a dataflow, the data can be routed to the sink successfully if there is a safely routed path in another dataflow topology. However, the deficiencies in this type of scheme are that: (a) the ability of this scheme to resist attacks is limited [3], and the scheme does not have the ability to identify compromised nodes; and (b) the energy consumption is larger, several times that of a single dataflow topology method [3].
(3) The scheme not only can detect whether there is an SFA but also can identify malicious nodes. The main point is to first detect whether there is an SFA in the network; if there is, the scheme identifies the malicious nodes by adopting an ACK-based mechanism. Then, some action will be adopted to eliminate the malicious nodes. Xiao, Yu *et al.* [5] have proposed a CHEMAS (checkpoint-based multi-hop acknowledgement scheme) to defend against SFA, and its details can be found in Section 4.1.

Based on the execution characteristics of SFA detection schemes, such schemes can be divided into distributed detection strategies and central strategies. In a distributed detection scheme, the source nodes, nodes in the routing path and the neighbor nodes are involved in monitoring and detecting whether an SFA exists. The detection results are summarized by the source node, and then the detection result can be reported to the system (or sink) [3]. In a centralized detection strategy, there is a center of information processing (sink); for example, the monitored information is reported to the information processing center [3,5]. In general, the distributed strategy has good performance, and the central strategy has a shortage of single-point failures [3,5].

According to different situations regarding the attacker-dropped packets, several cases can be considered: (a) a simple form of this attack is that a malicious node drops all the packets passing through it, *i.e.*, the malicious node acts like a black hole. This type of attacker is relatively easy to detect and is relatively rare as an SFA; (b) a more refined variant of this attack is when a malicious node selectively drops/forwards packets. This attacker is more intelligent and has a stronger disguise and a more deceptive nature [3,4,5]; it can drop packets of some specified nodes or drop packets of some specified type. This makes detection of the attack more complicated. In practice, the attacker tends to be very smart and strong, and it is more meaningful to research such attackers. Therefore, in this paper we assume that the attacker is very intelligent and has strong capabilities.

## 3. The System Model

### 3.1. The Network Model

(1) We consider a wireless sensor network consisting of a large number of sensor nodes that are uniformly and randomly scattered in a circle network; the network radius is *R*, with the density of nodes equal to ρ, and nodes do not move after being deployed [15,16,17,18]. On detecting an event, a sensor node will generate messages, and those messages must be transmitted to the sink node [12,13]. However, the routing method used for the data packets is determined based on the requirements of the application, such as the shortest routing approach [10,11].
(2) The attacker is considered to have strong intelligence [5]. It obtains legal identification through compromising a sensor node. After that, the attacker can launch various attacks, such as dropping data packets or ACK messages or altering messages with a certain probability. The aim of attackers is to try not to expose themselves and to cause the greatest harm to the network. At the same time, the attackers can also collude to launch attacks.
(3) A message authentication code is adopted in the PHACK scheme, which provides assurance to the recipient that the message came from the expected sender and has not been altered in transit [10]. Therefore, in this paper, if there are no special instructions, all packets or messages adopt the message authentication code technology. To facilitate the discussion, the contents of messages or packets can be given, and specific authentication technology can be found in [10].

### 3.2. Energy Consumption Model and Related Definitions

The typical energy consumption model is adopted [16,17], as reflected in the energy consumption for sending data in Equation (1) and for receiving data in Equation (2):

(1) $$\left\{ \begin{array}{l} E_{t}=lE_{elec}+l\varepsilon_{fs}d^{2}\;if\;d<d_{0}\\ E_{t}=lE_{elec}+l\varepsilon_{amp}d^{4}\;if\;d>d_{0} \end{array}\right.$$

(2) $$E_{r}(l)=lE_{elec}$$

$E_{elec}$ in the formula represents the energy consumption of the transmitting circuit. If the transmitting distance is less than the threshold $d_{0}$, the consumption of power amplification adopts the free space model. If the transmitting distance is more than the threshold $d_{0}$, it adopts the multipath attenuation model. $\varepsilon_{fs}$ and $\varepsilon_{amp}$ are the energy required to amplify power in the two models. l denotes the number of bits of data. In this paper, the parameter of the specific configuration above references [16,17], as shown as Table 1.

**Table 1:** Network parameters.

Parameter	Value
Threshold distance (*d*_0_) (m)	87
Sensing range *r_s_* (m)	15
*E_elec_* (nJ/bit)	50
*e_fs_* (pJ/bit/m^2^)	10
*e_amp_* (pJ/bit/m^4^)	0.0013
Initial energy (J)	0.5

## 4. The Design of the Protocol

### 4.1. Research Motivation

**Figure 1:**
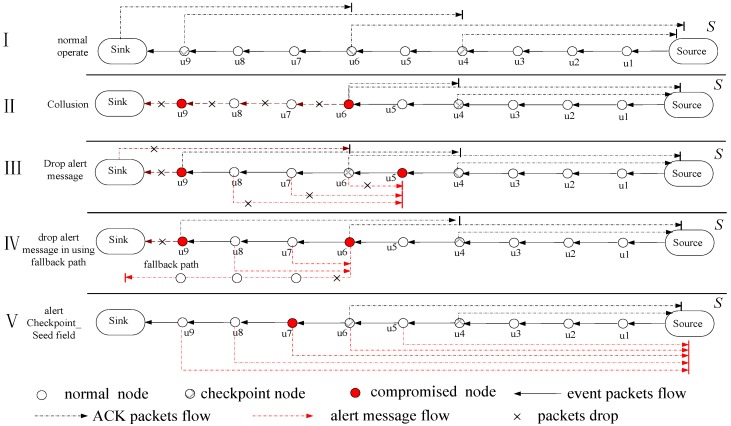
Overview of acknowledgment-based selective forwarding attack detection.

Acknowledgment-based selective forwarding attack detection is one of the more prominent approaches [5]. Its operation mechanism is shown as I in Figure 1, where a certain number of nodes is selected as checkpoint nodes in the routing path from the source node to the sink in the ACK-based scheme. As long as a checkpoint node receives a data packet, it will return an ACK to the upstream node. The ACK message contains the survival time for the ACK, that is, the time to live (TTL). The value of TTL minus 1 is when an ACK passes one checkpoint node. If the value of TTL in an ACK message is 0, then the ACK message is discarded. After the data are forwarded by the node, it then waits for the arrival of the ACK packet. If the node has not yet received the expected number of ACK messages, it will send an alert message to the source node. For example, consider Figure 1: when using TTL = 2, node u1 to u8 will receive 2 ACK packets. If some nodes do not receive 2 ACK packets, they will send an alert message to node *S* to warn that there is the behavior of a selective forwarding attack.

However, acknowledgment-based selective forwarding attack detection schemes have some deficiencies: (1) when more than two compromised nodes are elected as checkpoint nodes, the scheme will fail. For example, consider II in Figure 1: if the compromised nodes u6 and u9 are selected as checkpoint nodes, then u6 can attack the routing path by colluding with u9. When node u6 drops a data packet, it forges the ACK packet of u9 and sends the produced ACK by itself to the source node. This attack behavior cannot be detected by the SFA detection mechanism in this way; (2) Kim *et al.* [18] have noted that the ACK message and alert message are returned along the original data routing path, so the ACK packet and alert message can also be discarded by compromised nodes with a certain probability. This can also result in protocol failure [14]. This is shown by III in Figure 1: if u5 and u9 are compromised nodes, then the data packet can be discarded by node u9 and all alert messages can be discarded by u5 such that the source node does not receive any alert messages. This situation makes the source node think that the data packet reached the sink successfully, and no further action is adopted to resend the data, leading to the failure of this scheme; (3) If the collected alert messages by the source node are transmitted to the sink along the original data routing path, as long as there is a malicious node in the routing path, which can ensure that the source node cannot receive alert messages, the protocol is invalid. Kim *et al.*, proposed a scheme by which the alert message is transmitted to the sink along a fallback path to solve the above problems [18]. The improvement of [14] is that alert messages are transmitted to the sink along a fallback path after being merged by a checkpoint node. Therefore, if two selected checkpoint nodes are compromised nodes, the improved protocol can also be disabled. This can be observed from IV in Figure 1: if u6 and u9 are compromised nodes, then u9 discards the data packet and u6 discards all the alert messages. This protocol can also be defeated; (4) More seriously, some protocols think that compromised nodes can change the data of the Checkpoint_Seed field [5]. Thus, as long as any one node in the routing path has changed the data of the Checkpoint_Seed field, the selected checkpoint node in the downstream may not be correct. Though those selected checkpoint nodes can produce ACK packets, the ACK packets can be discarded because those ACKs are not produced by the correct checkpoint. This leads to nodes who have not yet received the expected number of ACK messages producing alert messages to create system confusion. This can be seen in V in Figure 1: if node u7 is a compromised node, it changes the data of the Checkpoint_Seed field. This leads to ACK packets of downstream nodes of u5 not being able to be produced by the right checkpoint node, which causes those ACK messages to be discarded by normal nodes, resulting in all the nodes in the routing path from u5 to the sink sending alert messages to the sink due to having received less than the expected number of ACK message. In this case, the problem cannot be solved by adopting the proposed improved scheme of Kim *et al*., which requires that all alert messages be securely transmitted to the sink [18]; (5) In the end, if the malicious nodes jointly attack the network using the above method, it will make the acknowledgment-based selective forwarding attack detection schemes more chaotic, thus making the protocol invalid.

The reasons for the deficiencies of the acknowledgment-based selective forwarding attack detection scheme are: (1) the ACK message and alert message are forwarded along the original data routing path; If all the data are normal on the routing path, the running of the protocol is very smooth, however, as long as there is a malicious node on the data routing path, the malicious node can attack said routing path, which disables the protocol; (2) the number of ACK messages is too few to locate malicious nodes accurately. To save energy, the previous research studies generate the minimal number of ACK messages, which are generated by only the checkpoint node. If the source node did not receive the expected ACK messages of the checkpoint, it cannot locate malicious nodes accurately.

### 4.2. Overview of the Proposed Scheme

**Figure 2:**
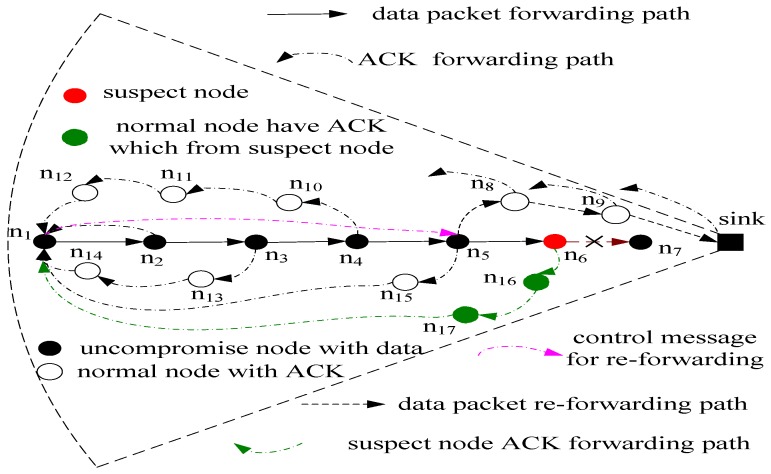
Illustration of the PHACK scheme.

To overcome the shortcomings of the SFA detection scheme, the PHACK scheme has improved the following three aspects:

(1) Every node returns an ACK to the sink when receiving a data packet. The aim is to overcome the shortcoming of too few ACK messages in the previous scheme so the locations of malicious nodes can be located more accurately. This can be observed from Figure 2. The data packets are routed from the source noden_1_ and then are routed along the routing path of n_1_→n_2_→n_3_→n_4_→n_5_→n_6_→n_7_→sink; each node in this path can return an ACK to the source node when receiving a data packet.
(2) The most important improvement of PHACK is that each ACK is forwarded along a routing path that is different from the original data routing path. Because each ACK is independently forwarded along a different routing path, it is difficult for malicious nodes to block these ACK packets. In the previous research studies, all the ACK messages are forwarded along the original data routing path. As long as the routing path has a malicious node, this scheme can be disabled. In the PHACK scheme, each produced ACK is forwarded along a different routing path. The ACK originating in noden_3_ is forwarded to the source node along noden_13_ and noden_14_ (see Figure 2). The ACK originating in node n_4_ is forwarded to the source node along noden_10_, noden_11_ and node n_12_.
(3) A re-routing mechanism is used, *i.e.*, when the data routing is blocked, the data do not need to be rerouted from the source node but instead from a normal node, that retains the data packet and is the nearest to the sink. This can be observed from Figure 2. When the source node receives an ACK from node n_6_ and does not receive an ACK from a node that is nearer to the sink than node n_6_, the source node can confirm that the data packet reached node n_6_. Thus, node n_6_ may be a malicious node. The reason for this can be explained as follows: (a) node n_6_ is likely a malicious node. The data packet can be dropped by noden_6_, but it also sends an ACK to the source node to the confused detector; (b) Node n_6_ is also likely a normal node. The data packet can be dropped in the following routing. However, in the PHACK scheme, the source node informs node n_5_ to reroute the data packet by sending control information, namely, the data packet can be rerouted along the routing path of node n_5_→n_8_→n_9_→sink. Compared with previous schemes, the advantage of the PHACK scheme is that the rerouted data packet does not need to be routed from the source node but instead from the node nearest to the sink, which can conserve network energy and cost. To the best of our knowledge, this is the first proposal of such a data rerouting mechanism.

### 4.3. The Format of Packets and Messages

**Figure 3:**
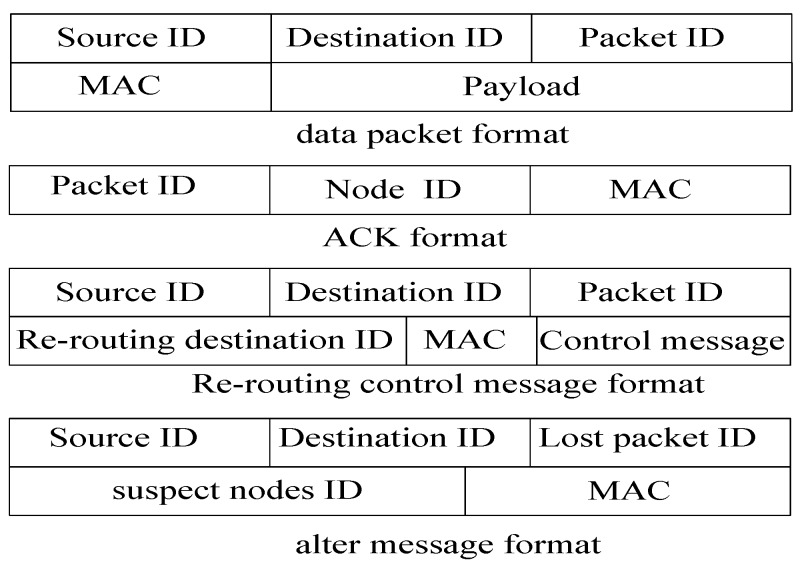
Packet format.

In the PHACK scheme, there are four different types of information flow. Its format is shown in Figure 3: (1) Data flow. It is generated by the source node, which contains five fields as shown in Figure 3. MAC stands for Message Authentication Code; (2) ACK flow. Each node generates an ACK packet when receiving a data packet, whose content includes the received packet ID, its own node ID, and the MAC. It can be used to identify the arrival location of the data packet routing. If the source node receives an ACK that is generated from a node, it can confirm that the data packet was routed to this node; (3) Control message for re-routing. It is used to assign a specified node for rerouting. The control message contains the suspect node ID. The suspect node cannot be selected as the next-hop routing node. In the PHACK scheme, each node stores the data packet for a period of time after the data packet is routed to the next node, which ensures that the data packet does not need to be rerouted from the source node to conserve network energy and reduce the routing delay. The time for a node to store a data packet is three times the time for a data packet to be routed to the sink. This is because if the node does not receive a re-routing message in this time, it means the data packet has been routed to the sink successfully. Thus, the node does not need to store the data packet any longer. The requirement of storage can be found in our previous work in [7]. We demonstrate that the storage for data is feasible due to the short time duration for the storage of data packets, and thus the system is available to the application; (4) Altered message. In the process of sending data to the sink, when the source node marks some nodes as suspect nodes according to the situation of the returned ACK packet, it reports the altered message to the sink. After the sink receives that altered message, it makes a decision or takes action to remove the suspect node.

### 4.4. Routing in the PHACK Scheme

In the PHACK scheme, there are four types of information flow routing mechanisms: (1) Data flow routing. Its routing scheme is adopted as the application routing scheme, such as shortest routing. The suspect node cannot be selected as the next node in the routing path when the data packets are routed to the sink; (2) Re-routing message routing. This message is used to indicate which node should start to re-route; (3) Altered message routing. Its routing is relatively simple. The successfully data route path is selected as its route path. Because the data packet has been successfully routed to the sink along this routing path, the altered message has a higher success rate along this path; (4) ACK flow routing. The formation of ACK flow routing is shown in Figure 2. The formation process is as follows: the nodes that generate an ACK random walk κ hop in the direction away from himself, and then the ACK is routed to the source node using the shortest routing scheme. For the specific algorithm, please see Algorithm 1.
**Algorithm 1** The ACK Routing Mechanism**Initialize:** If node a receives a data packet:
1:Node a produces an ACK message ξ and random number κ2:**While** κ > 0 Do3:The node that holds ξ forwards it to the neighbor node that is farthest from the source node 4:κ = κ − 1;5:End while6:**While the node isnot a source node** Do7:**Send** ξ to the node that is nearest to the source node8:**End while**

### 4.5. Identification of Suspect Nodes

Because each node returns an ACK when receiving a data packet, identifying suspect nodes becomes easier in the PHACK scheme. There are mainly two types of suspect nodes: (1) the last node for the return of the ACK; and (2) the last downstream node for the return of the ACK. It may be a malicious node is the last node for returning the ACK because the node may return an ACK when receiving a data packet. The next-hop node of the last node for returning the ACK may also be a malicious node. Because the data can be dropped and no ACK is returned by this node, if the source node does not receive an ACK, the last node and the next hop node can be marked as suspect nodes, and the situation is reported to the sink through an alter message. The sink makes decisions through comprehensive analysis of the received information.

## 5. Performance Analysis and Optimization

### 5.1. Energy Consumption and Network Lifetime

Considering the network radius *R = mr*, the hop count between the source node *S* and the sink is h, and the proposed scheme in this paper will not affect the network lifetime.

**Theorem 1.** *Considering the network radius is R = mr and the density of nodes is ρ, the average energy consumption of the nodes in the ith ring of the PHACK scheme is:*

(3) $$\lambda e_{u}\left(\frac{\left(m^{2}-i^{2}\right)}{\left(2i-1\right)}+\frac{\left(1+\varepsilon )i(m^{2}-i^{2}\right)}{k(2i-1)}+1\right)$$

**Proof.** Considering that the length of the data packet is l bits and the length of the ACK is δ bits, set *k* = *l*/δ. The event production rate is λ, and the number of data loads of nodes is as follows:

The data packets produced by the nodes in the *i*th ring and the >ith rings must pass the nodes in the *i*th ring. The number of nodes in the ≤*i*th rings is $\pi {\left(ir\right)}^{2}\rho$, and the number in the entire network is $\pi m^{2}r^{2}\rho$. It can be computed that the number of nodes in the >*i*th rings is $\pi m^{2}r^{2}\rho \;-\;\pi i^{2}r^{2}\rho$. The number of data packets loaded by the nodes in the *i*th ring is $\pi r^{2}\rho \lambda (m^{2}-i^{2})$.

The energy consumption for forwarding a packet is $e_{u}$, while the total number of nodes in the *i*th ring is:

(4) $$\pi i^{2}r^{2}\rho -\pi {\left(i-1\right)}^{2}r^{2}\rho \;=\;\pi (2i-1)r^{2}\rho$$

Thus, the energy consumption of the nodes in the ith ring for forwarding the data from the nodes in other rings is:

(5) $$\frac{\pi r^{2}\rho \lambda (m^{2}-i^{2})e_{u}}{\pi (2i-1)r^{2}\rho }\;=\;\frac{\left(m^{2}-i^{2}\right)}{\left(2i-1\right)}\lambda e_{u}$$

At the same time, the energy consumption of forwarding the data packets of the nodes in the *i*th ring is $e_{u}\lambda$. The average energy consumption for forwarding those data packets is $e_{u}\lambda$+$\lambda e_{u}(m^{2}-i^{2})/\left(2i-1\right)$.

In the PHACK scheme, each node returns an ACK when receiving a data packet, and then the nodes in the ithring have to load the ACK from the nodes in the ≥ *i*th rings. The number of loaded routing paths by the nodes in the *i*th ring is $\pi r^{2}\rho \lambda (m^{2}-i^{2})$. The produced ACK packets by every routing path need to be forwarded by the nodes in the *i*th ring. The number of returned routing paths for transmitting an ACK is *i*, so the nodes in the *i*th ring load the following number of ACK packets:

(6) $$i\pi r^{2}\rho \lambda (m^{2}-i^{2})(1+\varepsilon )$$

where ε is a factor of increasing routing path times when an ACK packet is not returned along the original path, so the number of ACK packets by each node in the *i*th ring can be obtained via: $i\left(1+\varepsilon )\lambda e_{u}(m^{2}-i^{2}\right)/\left(k(2i-1)\right)$. Thus, Equation (3) can be obtained.


 
■

**Conclusion 1.** *Compared to other acknowledgment-based schemes, the PHACK scheme does not affect the network lifetime.*

**Proof.** Only considering the energy consumption of the routing data, it is obvious that the nodes in the first ring in any acknowledgment-based scheme must load the returned ACK by the sink when *i* = 1, and the nodes must return one ACK to the source node, so the energy consumption in the first ring in the PHACK scheme is the same as that of other acknowledgment-based schemes. Theorem 1 has proved that the total average energy consumption of the nodes in the first ring is Equation (3). Thus, when *i* = 1, the energy consumption is:

(7) $$E_{1}\;=\;\lambda e_{u}\left(m^{2}+\frac{\left(1+\varepsilon )(m^{2}-1\right)}{k}\right)$$

If we can prove that, for all the nodes in i≥2nd rings, there is $E_{i}\;<\;E_{1}$, then the conclusion can be obtained that the PHACK scheme will not affect the network lifetime. That is to say, the following equation must be proved.

(8) $$\lambda e_{u}\left(\frac{\left(m^{2}-i^{2}\right)}{\left(2i-1\right)}+\frac{\left(1+\varepsilon )i(m^{2}-i^{2}\right)}{k(2i-1)}+1\right)\leq \lambda e_{u}\left(m^{2}+\frac{\left(1+\varepsilon )(m^{2}-1\right)}{k}\right)|i\geq 2$$

because in Equation (4), when i≥2, the two equations $\frac{\left(m^{2}-i^{2}\right)}{\left(2i-1\right)}+1<m^{2}$ and $\frac{\left(1+\varepsilon )i(m^{2}-i^{2}\right)}{k(2i-1)}<\frac{\left(1+\varepsilon )(m^{2}-1\right)}{k}$ are obtained. Thus, Equation (4) was established.


 
■

**Figure 4:**
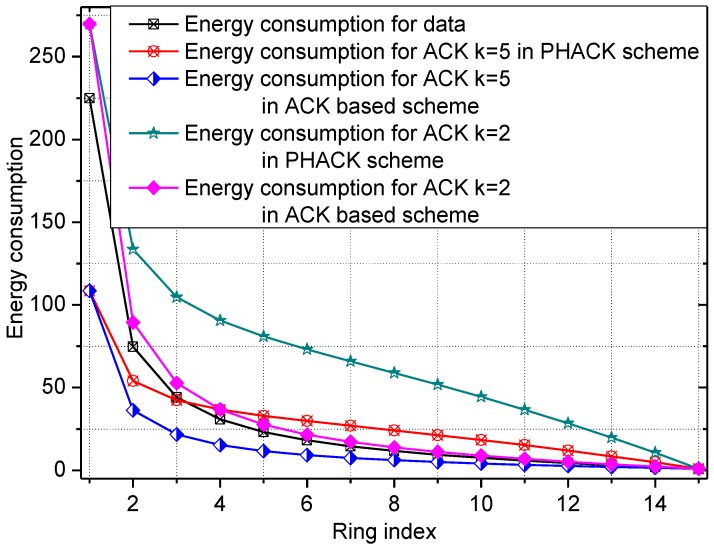
The energy consumption for data and ACKs.

**Figure 5:**
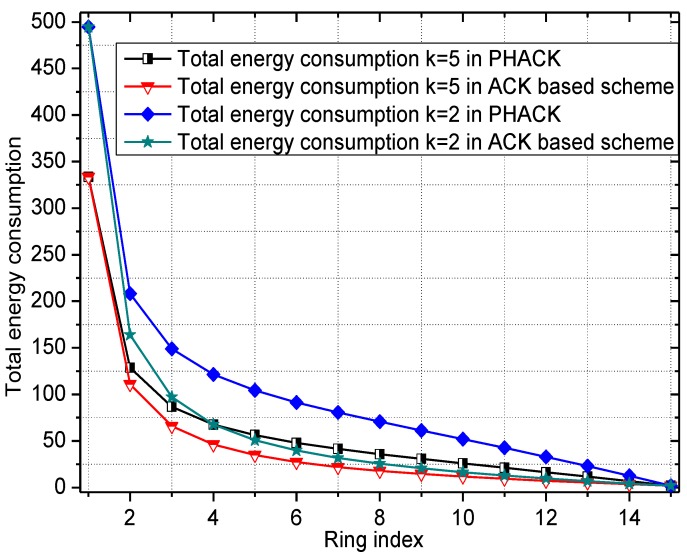
The total energy consumption in different schemes.

The energy consumption of the network in the PHACK scheme and an ACK-based scheme are given in Figure 4 and Figure 5, respectively. The energy consumption for transmitting data packets in the PHACK scheme is the same as for the ACK-based scheme (see Figure 4). The energy consumption for transmitting ACK messages in the PHACK scheme is the same as for the ACK-based scheme in hotspot areas, but it is larger in non-hotspot areas (see Figure 4). The total energy consumption of hotspot areas in PHACK is the same as for the ACK-based scheme, but it is larger in non-hotspot areas (see Figure 5). Because the lifetime is defined as the first node die time, the PHACK scheme does not affect the network lifetime.

### 5.2. Attack Analysis

In the PHACK scheme, the ACK generated by the nodes will be returned to the sink along different routing paths, which makes the scheme simpler and easier to implement; moreover, the security performance in this scheme is higher than all other proposed schemes:

(1) First, the PHACK scheme has a strong ability to resist attacks. The MAC and data ID of each node can be coded by a digital signature to form an ACK message and report whether the node received the data packet to the source node along different routing paths. Therefore, the node that only receives a data packet can send an ACK to the source node, so a malicious node can also produce an ACK packet when it receives a data packet. In the PHACK scheme, for a malicious node, there are four methods to damage the network: (a) the node does not produce an ACK packet and drops the data when receiving a data packet. In this case, the source node does not receive an ACK from this node and the following nodes, and thus this node is a suspect node. Thus, the malicious node is invalidated; (b) the node does not produce an ACK packet when receiving a data packet, but it forwards the ACK produced by other nodes to the source node. In this case, because the downstream nodes return an ACK packet to the source node when receiving a data packet, this node may be marked as a suspect node when the ACK of this node is intercepted; (c) The node returns an ACK and drops the data when receiving a data packet. Because the node is the last node to return an ACK packet to the source node, this node may also be marked as a suspect node; (d) the node returns an ACK and forwards the data packet when receiving a data packet. This type of situation is the same as that of a normal node and does not harm the network, and thus it is consistent with the aim of protecting network security.
(2) In the PHACK scheme, a node cannot fabricate alert packets. In previous schemes, whether a node generates an alert packet is determined by itself. Thus, any node can generate false alert packets or fabricate alert packets. In the PHACK scheme, a node generates an alter packet under the condition that the nodes have received a data packet. Thus, the situation in which a compromised node fabricates alert packets maliciously, resulting in the prosecution of innocent normal nodes, cannot occur [5].
(3) Higher success rate of data transmission. In a network with a higher percentage of malicious nodes, there may be multiple malicious nodes in the routing path. These malicious nodes may drop data and ACK data alone or work together to drop both data and ACK data. Because all the data and the ACKs are routed in the same routing path in previous schemes, this leads to the invalidation of the scheme. Though those schemes have the ability to detect an SFA in theory, they cannot meet the requirements of applications due to data being unable to arrive at the sink in time. In the PHACK scheme, each ACK is independently routed to the sink along different routing paths, even if the malicious node can block most of the ACK messages. As long as one ACK is routed to the source node successfully, it will be able to indicate the reached location of the data packet. That is to say, the probability of malicious nodes blocking the routing is reduced by several times. More importantly, a re-routing mechanism is adopted in the PHACK scheme, so the failed routing can be re-routed in the interception node. The data need not be retransmitted from the source node, therefore ensuring not only a higher success rate but also the shorter routing time.

### 5.3. Detection Probability Analysis

For the sake of simplicity, the network is assumed to operate under ideal radio conditions; consequently, the dropping of packets is the only reason for packet loss. There are *n* sensor nodes in the entire network, *m* of which are malicious nodes. We suppose that the source node is ℏ hops from the sink, so there are c=ℏm/n malicious nodes in the forwarding path to the sink.

(1) The probability of malicious nodes dropping data. In the PHACK scheme, as long as the data packet is dropped, the source node will not receive an ACK from the sink. Thus, the dropped packet can be detected as long as it is dropped. That is to say, the probability of detecting the packet loss is 1 in PHACK. In previous schemes, if the data packet is dropped, the alter message is returned by the original path. Thus, as long as there is a malicious node in the return path, the source node will not be able to detect that the data are dropped. Considering the return path length of later messages is ∂ℏ hops, statistically there are *b* = ∂ℏm/n malicious nodes in that path. Considering that malicious nodes drop alter messages with a random probability of τ, an alter message can reach the source node if all malicious nodes refrain from dropping said alter message. The probability of the detection of dropped datain the ACK-based scheme is:
  (9) $$p_{d}^{1}\;=\;1\;-\;{\left(1-\tau \right)}^{b}|b\;=\partial \hbar m/n$$

**Figure 6:**
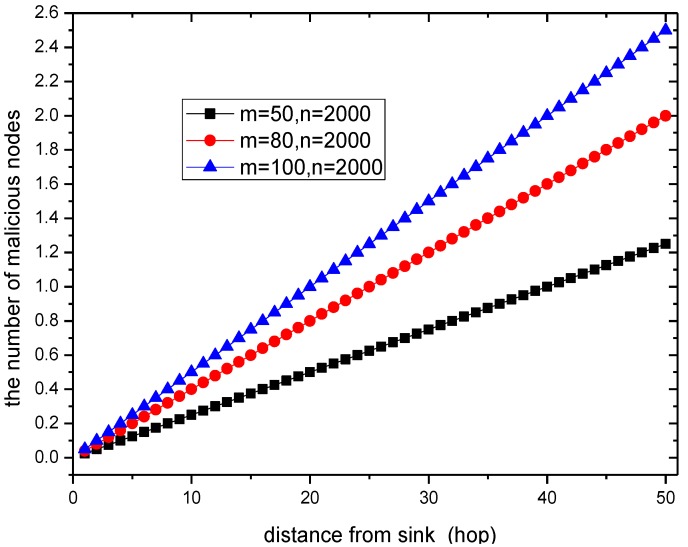
The number of malicious nodes in a routing path.

When the number of nodes deployed in the network is 2000 and the malicious nodes are 50, 80, or 100 strong, the number of malicious nodes in the routing path from the node with different distance to sink is given in Figure 6. 

**Figure 7:**
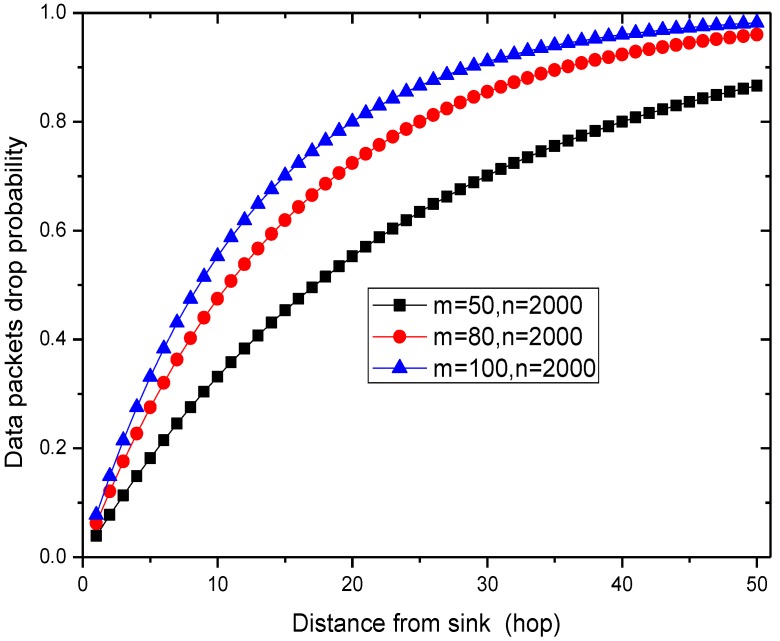
The data drop probability.

When the ratio of malicious nodes and all nodes in the network is 50/2000 = 2.5%, the number of malicious nodes is more than 1 in the routing path from the node that is a distance of 50 hops from sink to sink. Thus, the probability that the network is attacked is high, which can also be seen from Figure 7. However, in previous schemes, the probability that the data packet is dropped and is not detected is as high as $p_{d}^{1}$. Those packets cannot arrive at the sink. However, in the PHACK scheme, the probability of detecting the packet loss is 1, which has significant meaning for ensuring network security.

(2) The probability and the number of times for data packet retransmission. It refers to the situation in which the data packet needs to be retransmitted when the source node does not receive the data packet. In the PHACK scheme, in the process of routing, as long as an ACK reaches the source node, it does not need to be retransmitted. The probability for this situation is: $p_{d}^{2}=\tau m/n$. However, in the ACK-based scheme, the probability is $p_{d}^{3}=\tau \hbar m/\left(2n\right)$. Thus, the probability in the PHACK scheme is reduced by ℏ/2 times.

## 6. Experimental Results

OMNET++ is used for experimental verification [19]. In the experiment, the setup is as follows: the network radius *R* = 400, there are 1000 nodes in the network, there are 100 malicious nodes, and the probability of malicious nodes dropping data of malicious nodes is 0.7. The ratio of packet length by ACK over data packet is 1/5. In one round, each node generates a data packet that should be transmitted to the sink node by using the shortest routing approach [6,7]. The ACK can be returned to the source node along a different routing path when a node receives a data packet. After a malicious node is identified, this node will be replaced by a normal node. At the same time, a normal node can be changed into a malicious node to maintain the ratio of malicious nodes.

**Figure 8:**
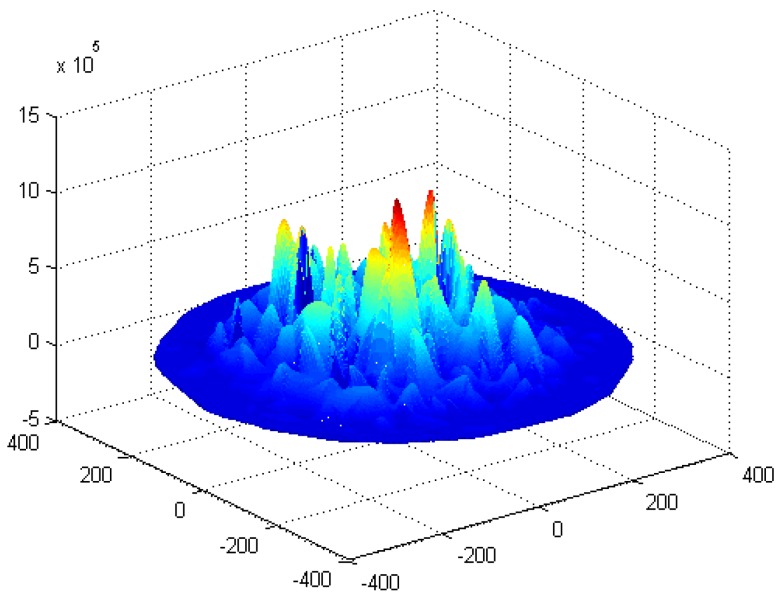
The energy consumption in the PHACK scheme.

**Figure 9:**
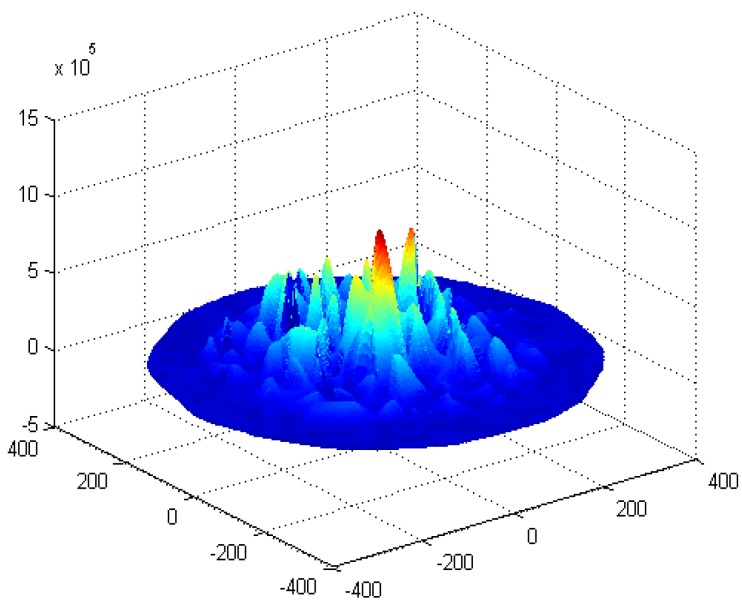
The energy consumption in the ACK-based scheme.

The energy consumption of the network in the PHACK scheme and ACK-based scheme is given in Figure 8 and Figure 9, respectively. It can be observed that the energy consumption of the PHACK scheme is more balanced than that of the previous schemes. This can be explained as follows: (a) in the PHACK scheme, the nodes in hotspots do not send ACKs for received data packets; instead, the sink returns ACKs for each received data packet. This is the same with the ACK-based scheme. Thus, if the received data packets of the nodes in hotspot areas in PHACK are the same as for the previous schemes, the number of ACK loads of nodes is also the same in hotspot areas; (b) in different schemes, the number of data loads of nodes in hotspot areas is the same if the loss probability of data packets is the same. If the loss probability of data packets is reduced due to the higher security in the PHACK scheme, the amount of data loads of nodes in hotspot areas will be increased, which causes the amount of sent ACKs to increase. This is the aim such that all data packets can arrive at the sink successfully. In summary, in the PHACK scheme, if the loss probability of data packets is the same as for the other schemes, the energy consumption of the nodes in hotspot areas is the same. If the loss probability of data packets is decreased, the amount of data loads of nodes will be increased. The reason is not that the scheme is different from the previous schemes, but instead that the arrival probability of data packets to the sink is higher due to the higher security. This shows that the PHACK scheme is better than the previous scheme; (c) In non-hotspot areas, each node forwards the ACK packet to the source node along a different routing path when it receives a data packet, increasing the energy consumption in the non-hotspot areas. It can be seen from Figure 9 that there is much more energy left in non-hotspot areas. From Figure 8, it also has much energy left in non-hotspot areas in the PHACK scheme. That is to say, although nodes return an ACK when they receive a data packet, there is much energy left in non-hotspot areas (which is consistent with Conclusion 1 in Section 5). However, less energy is left than in the previous scheme. Network lifetime can be defined as the time the node that dies first. The node with the largest energy consumption is in a hotspot area, so the network lifetime in the PHACK scheme is equal to that of the previous scheme. The decline of network lifetime is caused by the increased probability of data packets being transmitted to the sink successfully. Thus, we can conclude that the PHACK scheme does not affect the network lifetime.

**Figure 10:**
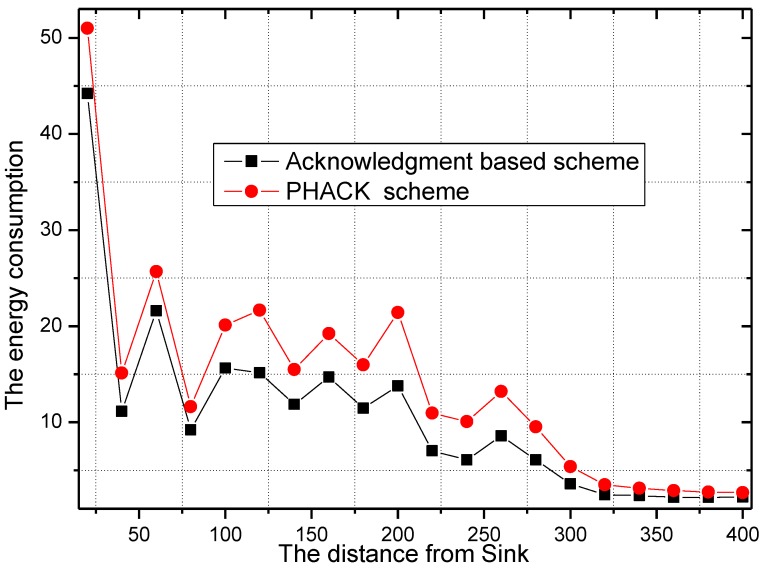
The energy consumption in different schemes.

**Figure 11:**
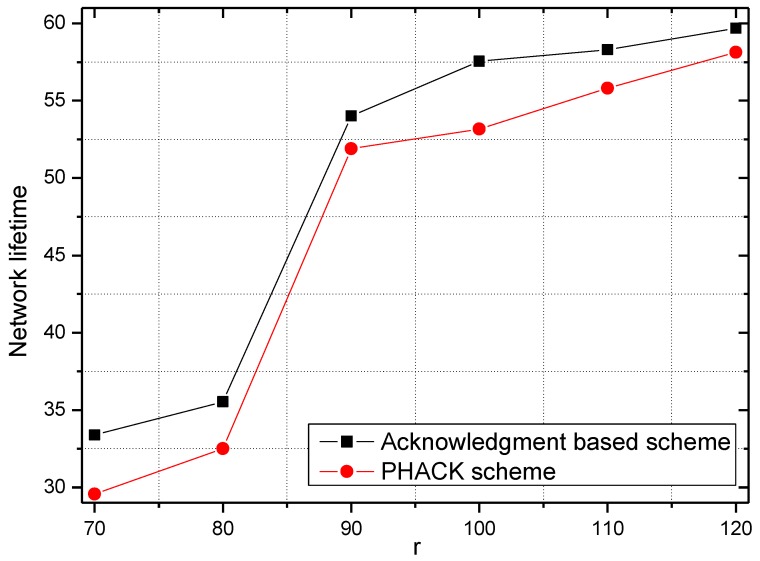
The network lifetime in different schemes.

The energy consumption and network lifetime under different schemes in the network are shown in Figure 10 and Figure 11, respectively. From Figure 10, the reason that the network lifetime decreases in the PHACK scheme is that the success probability of data packets being routed to the sink is increased and so the energy consumption in hotspot areas is increased. This can be observed in the following experimental results. The success rate of the PHACK scheme’s data packets is increased by 12%, so the decline of its network lifetime shows that the scheme is effective.

**Figure 12:**
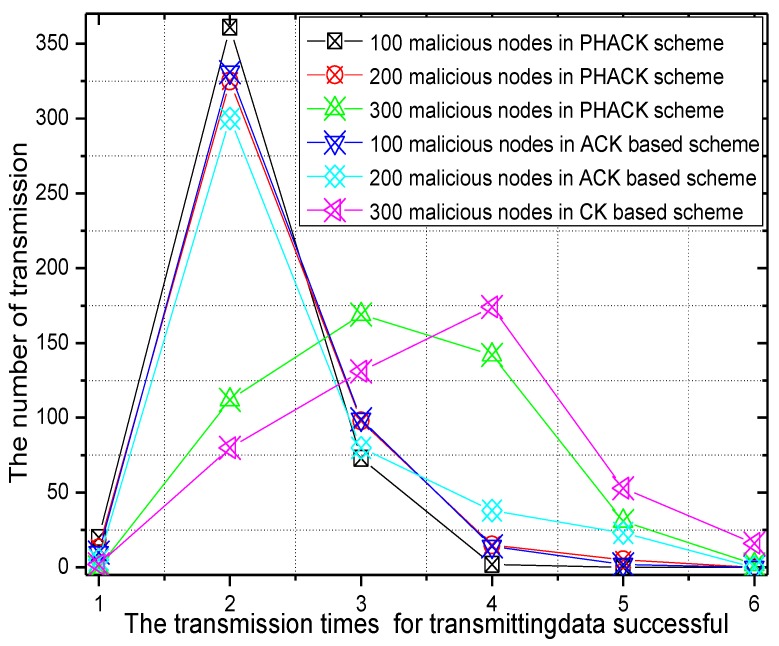
The transmission times for successful data transmission.

**Figure 13:**
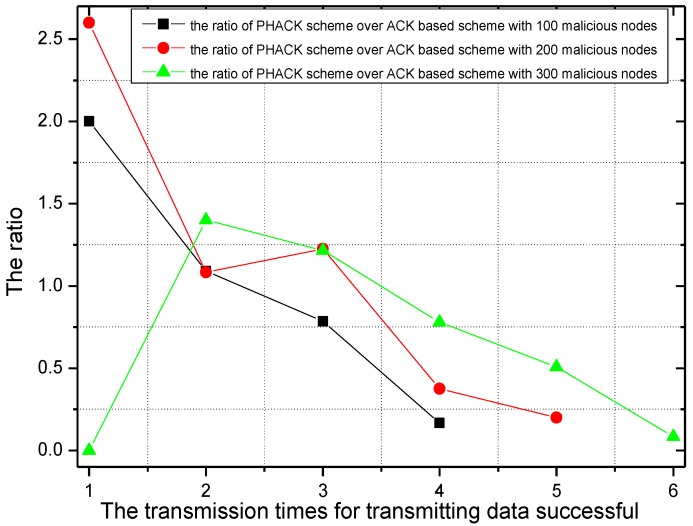
The ratio of transmission times for successful data transmissionof PHACK over that of the ACK-based scheme.

For different numbers of malicious nodes, the transmission times for successful data packet transmission and the rate of transmission times of the PHACK scheme over the ACK-based scheme are given in Figure 12 and Figure 13. In the experiment, there are 456 transmissions launched by the node that has the largest number of hops to the sink. When there are 100 or 200 malicious nodes in the network, *i.e.*, the percentage of malicious nodes in the network is small, the probability of packet loss is small, which means most of the data packets can be transmitted to the sink successfully with shorter transmission times. Approximately 350 of 456 transmissions can be sent to the sink successfully with only two transmissions (see Figure 12). However, if there are 300 malicious nodes in the network, that is to say, there are many malicious nodes in the network, the probability for data to be dropped is large, which leads to data packets reaching the sink after the data are rerouted several times. In this case, most data packets will not be transmitted successfully until they are transmitted three or four times (see Figure 12). It can be observed from Figure 13 that, in the PHACK scheme, the transmission times of the data packets of most source nodes are less, and the arrival probability of data packets in the PHACK scheme is two times more than that of the ACK-based scheme. This fully demonstrates the effectiveness of the PHACK scheme.

**Figure 14:**
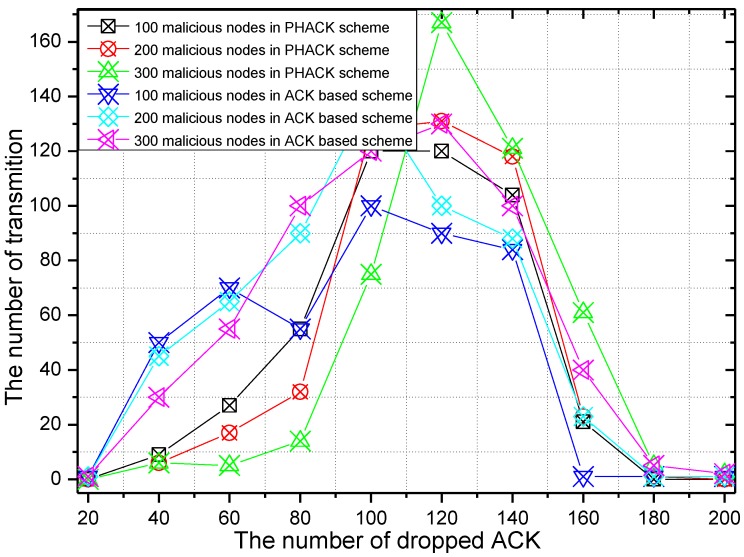
The number of dropped ACKs.

**Figure 15:**
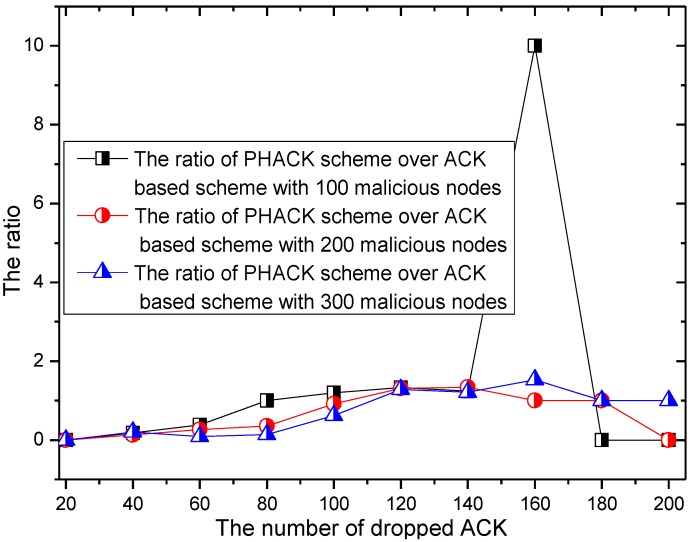
The ACK dropratio of PHACK over theACK-based scheme.

The number of dropped ACK packets and the rate of the dropping of ACKs of the PHACK scheme over the ACK-based scheme when the data packet is routed to the sink successfully are given in Figure 14 and Figure 15, respectively. It can be seen from Figure 14 that the trend is roughly the same with data packet transmission. The difference is that, with the increase of malicious nodes in the network, the number of dropped ACKs also increase. From Figure 15, in general, the number of dropped ACKs in the PHACK scheme is less than that of the ACK-based scheme.

**Figure 16:**
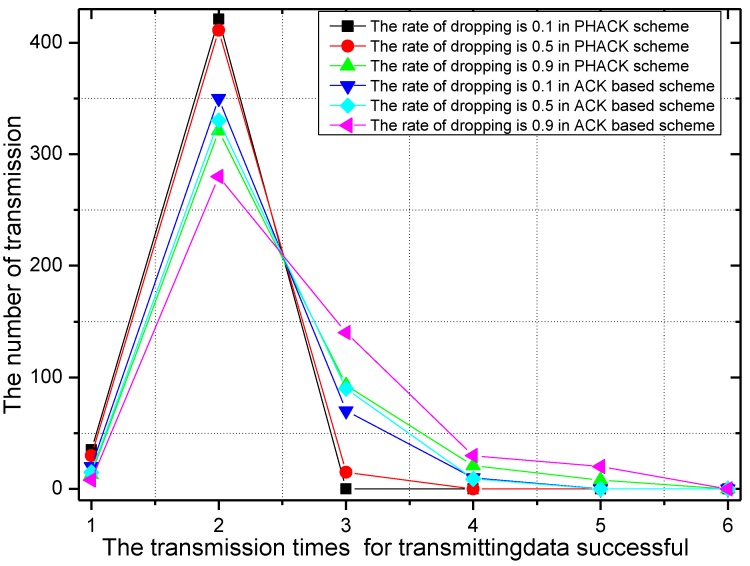
The number of successful data transmissions in the PHACK scheme with different rates for the dropping of data.

**Figure 17:**
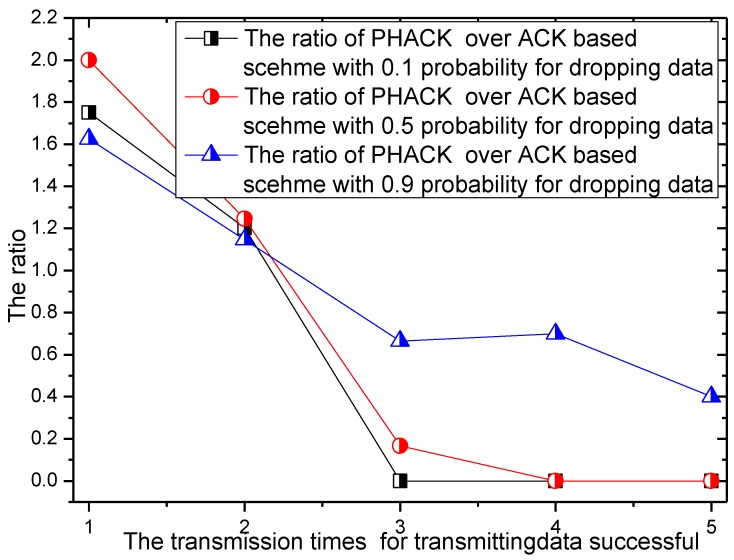
The ratio of PHACK and the ACK-based scheme with different rates for the dropping of data.

With different probabilities of the dropping of data, the required transmission times of the data packet and the rate of the required transmission times of the data packets in the PHACK scheme and ACK-based scheme are given in Figure 16 and Figure 17, respectively. It can be observed from Figure 16 that with the increase of the probability of the dropping of data, the required transmission time is increased when the data packet is transmitted to the sink successfully; the reasons for this is that, if the probability of the dropping of data increases, the probability of searching for other routing paths of the data packet is increased, so the transmission time of the data packet is increased. From Figure 17, most of the data produced by the source node can be transmitted to the sink on the second try in the PHACK scheme; those source nodes are approximately 1.2 times than that of the ACK-based scheme. This shows that PHACK has better performance.

**Figure 18:**
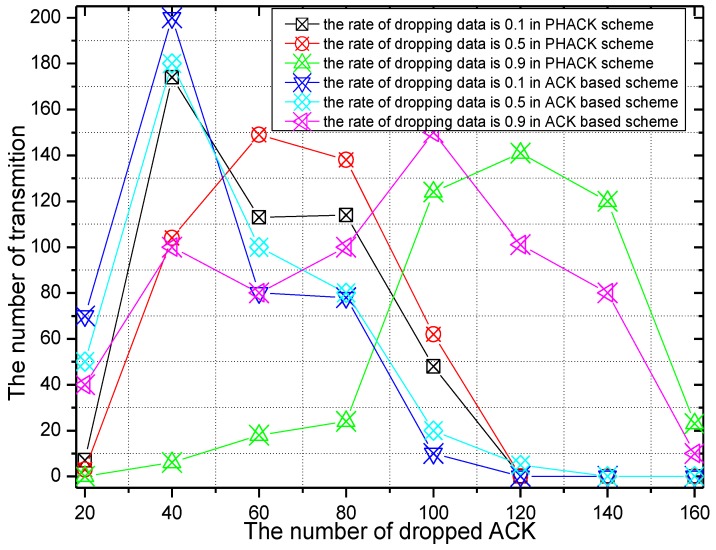
The number of dropped ACKs with different rates for dropping data.

**Figure 19:**
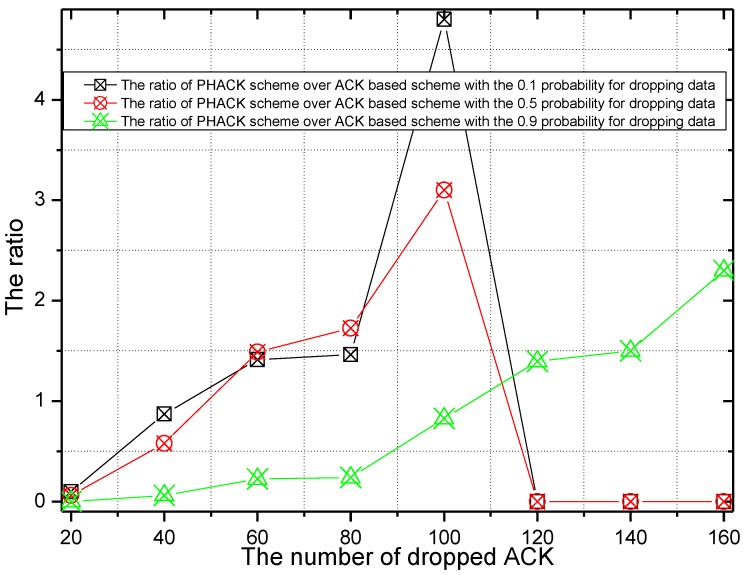
The ACK dropping ratio of PHACK over the ACK-based scheme.

The probability of malicious nodes dropping packets is set to 0.1, 0.5, and 0.9 in the experiment of Figure 18 and Figure 19. The number of dropped ACKs and the ACK dropping ratio of PHACK over the ACK-based scheme when the data packet is routed to the sink successfully are given in Figure 18 and Figure 19, respectively. It can be observed from Figure 18 that, with the increase of the probability of the dropping of data by malicious nodes, the maximum number of dropped packets in the network transmission is also increased. From Figure 19, when the probability of the dropping of data is smaller, such as 0.1 or 0.5, the dropped ACKs in the PHACK scheme are more numerous than those of the ACK-based scheme. The reason for this is that each node returns an ACK when it receives a data packet in the PHACK scheme. Because the number of generated ACK packets in the PHACK scheme is larger than that in the ACK-based scheme, the number of dropped ACKs is larger as well.

**Figure 20:**
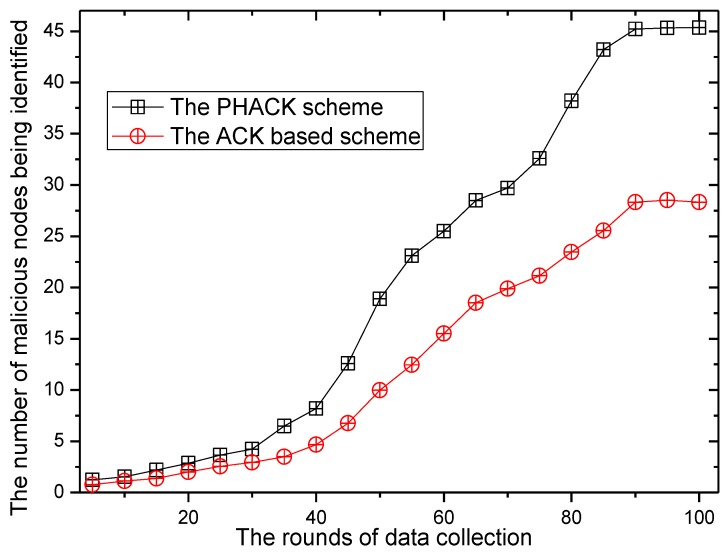
The number of malicious nodes being identified.

**Figure 21:**
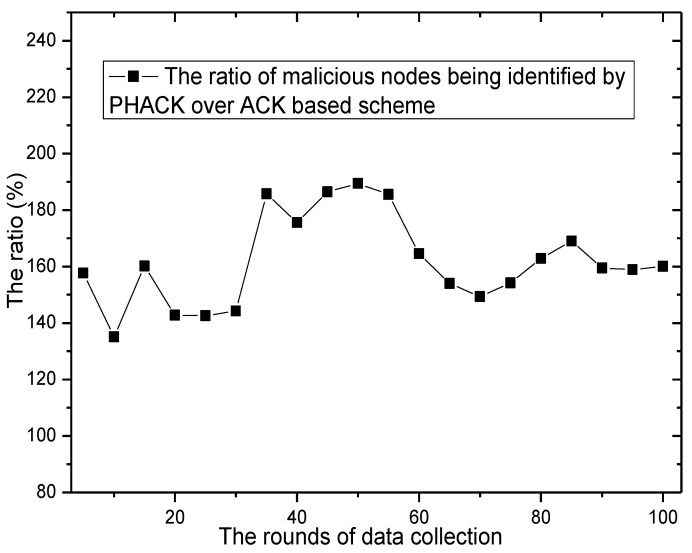
The ratio of malicious nodes being identified by PHACK over the ACK-based scheme.

The number of malicious nodes being identified can be found in Figure 20, where it can be observed that by running the scheme, the number of malicious nodes being identified is also increased. Additionally, the number of malicious nodes being identified in the PHACK scheme is greater than that of the ACK-based scheme. The ratio of malicious nodes being identified by PHACK over the ACK-based scheme is greater than 1.25 (see from Figure 21). This shows that the PHACK scheme has a stronger ability to identify malicious nodes, that is, it has higher security.

## 7. Conclusions and Future Work

In this paper, a simple and efficient PHACK scheme is proposed for detecting selective forwarding attacks and recovering the failed route. The main differences from previous research studies are: (1) the use of a selective forwarding attacks detection method in which the nodes can produce ACKs and return the ACKs to the source node along different paths when the nodes receive a data packet, which can simplify the scheme and improve the ability to locate malicious nodes and detecting an SFA. More importantly, it has been theoretically proved that although the PHACK scheme produces significantly more ACK packets than other schemes, it does not affect the network lifetime. Thus, it can avoid the defects of design complexity and low safety performance of selective forwarding attack detection in the scheme; (2) The PHACK scheme not only has the ability to detect a selective forwarding attack, but also can recover the routing from the location at which the data were dropped as soon as possible. This is of vital significance for ensuring the security of wireless sensor networks.

In our future work, we would like to explore additional mechanisms to ensure that our protocols continue to function even in the face of a powerful adversary that can collude with other attackers to launch attacks. In fact, selective forwarding attacks are widely used in multiple wireless networks. They not only exist in wireless sensor networks but also exist in crowd sensing networks. Moreover, due to the wide range of communication, the mobility of nodes, and multiple data collection centers in crowd sensing networks, the security is much more important for research. Thus, we would also like to study the security of crowd sensing networks to prevent selective forwarding attacks.
